# Alcohol Consumption and a Decline in Glomerular Filtration Rate: The Japan Specific Health Checkups Study

**DOI:** 10.3390/nu15061540

**Published:** 2023-03-22

**Authors:** Yoshiki Kimura, Ryohei Yamamoto, Maki Shinzawa, Katsunori Aoki, Ryohei Tomi, Shingo Ozaki, Ryuichi Yoshimura, Akihiro Shimomura, Hirotsugu Iwatani, Yoshitaka Isaka, Kunitoshi Iseki, Kazuhiko Tsuruya, Shouichi Fujimoto, Ichiei Narita, Tsuneo Konta, Masahide Kondo, Masato Kasahara, Yugo Shibagaki, Koichi Asahi, Tsuyoshi Watanabe, Kunihiro Yamagata, Toshiki Moriyama

**Affiliations:** 1 Department of Nephrology, Graduate School of Medicine, Osaka University, Suita 565-0871, Japan; kimukimu_y2k@infoseek.jp (Y.K.);; 2 Department of Nephrology, National Hospital Organization Osaka National Hospital, Osaka 540-0006, Japan; 3 Health and Counseling Center, Osaka University, Toyonaka 560-0043, Japan; 4 The Japan Specific Health Checkups (J-SHC) Study Group, Fukushima, Japan; 5 Health Promotion and Regulation, Department of Health Promotion Medicine, Osaka University Graduate School of Medicine, Toyonaka 560-0043, Japan

**Keywords:** alcohol consumption, dose-dependent association, epidemiology, glomerular filtration rate, retrospective cohort study

## Abstract

Previous studies have reported conflicting results on the clinical impact of alcohol consumption on the glomerular filtration rate (GFR). This retrospective cohort study aimed to assess the dose-dependent association between alcohol consumption and the slope of the estimated GFR (eGFR) in 304,929 participants aged 40–74 years who underwent annual health checkups in Japan between April 2008 and March 2011. The association between the baseline alcohol consumption and eGFR slope during the median observational period of 1.9 years was assessed using linear mixed-effects models with the random intercept and random slope of time adjusting for clinically relevant factors. In men, rare drinkers and daily drinkers with alcohol consumptions of ≥60 g/day had a significantly larger decline in eGFR than occasional drinkers (difference in multivariable-adjusted eGFR slope with 95% confidence interval (mL/min/1.73 m^2^/year) of rare, occasional, and daily drinkers with ≤19, 20–39, 40–59, and ≥60 g/day: −0.33 [−0.57, −0.09], 0.00 [reference], −0.06 [−0.39, 0.26], −0.16 [−0.43, 0.12], −0.08 [−0.47, 0.30], and −0.79 [−1.40, −0.17], respectively). In women, only rare drinkers were associated with lower eGFR slopes than occasional drinkers. In conclusion, alcohol consumption was associated with the eGFR slope in an inverse U-shaped fashion in men but not in women.

## 1. Introduction

Alcohol consumption is a major modifiable risk factor for the global health burden [1]. Systematic reviews reported a J-shaped association between alcohol consumption and all-cause mortality [2,3,4,5]. Alcohol consumption causes various health problems [6]: a J-shaped association has been reported with stroke [7,8], especially ischemic stroke [9,10]; a U-shaped association with type 2 diabetes [11,12,13,14]; a positive dose-dependent association with atrial fibrillation [15,16], heart failure [7], hemorrhagic stroke [9,10], breast cancer [17,18], and colorectal cancer [18,19]; and a negative dose-dependent association with ischemic heart disease [7,8,20].

The clinical impact of alcohol consumption on kidney function in the general population is controversial. Alcohol consumption has been associated with the incidence of end-stage kidney disease (ESKD) in a positive [21] or negative [22] dose-dependent manner. Another cohort study reported no significant association between alcohol consumption and the incidence of ESKD [23]. The incidence of chronic kidney disease (CKD), which is defined as a glomerular filtration rate (GFR) of <60 mL/min/1.73 m^2^, was associated with alcohol consumption in a negative dose-dependent [24,25,26,27] and U-shaped manner [28,29]. One of the main reasons for the different associations in previous studies might be the varied range of the highest alcohol consumption: >10–30 [24,25,26,27], ≥46 [30], ≥48 [31], and >69 g/day [28]. In order to assess the dose-dependent association between alcohol consumption and the GFR accurately, the trajectory of the GFR should be analyzed in heavy drinkers with an alcohol consumption rate of ≥60 g/day. Because previous studies have reported that women are more vulnerable to the deleterious effect of high alcohol consumption than men [30,31], the dose-dependent association between alcohol consumption and the GFR should be assessed in men and women separately.

This retrospective cohort study aimed to investigate the dose-dependent association between alcohol consumption and the GFR trajectory in a large cohort of the general population, including 304,929 participants (125,698 men and 179,231 women) who underwent annual health checkups in Japan. The findings of the present study suggest a potential threshold to prevent the deleterious effects of alcohol consumption when considering the GFR.

## 2. Materials and Methods

### 2.1. Participants

**Figure 1:**
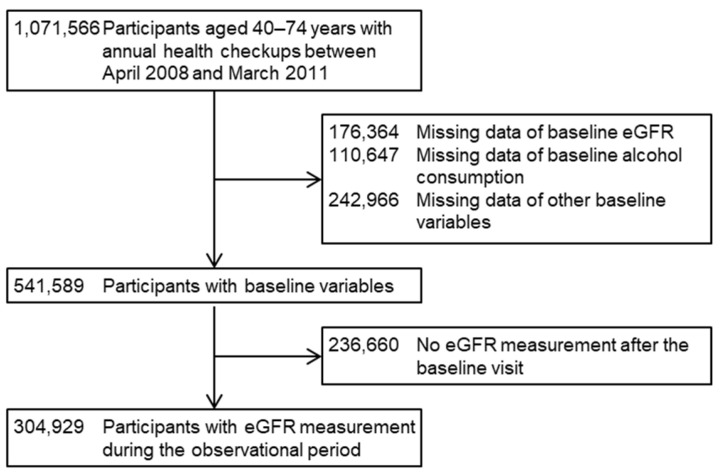
Flow diagram of inclusion and exclusion of the study participants. CVD, cardiovascular disease; eGFR, estimated glomerular filtration rate.

This study included 1,071,566 participants who were eligible for the study, aged 40–74 years and underwent their annual health checkups in 26 prefectures in Japan between April 2008 and March 2011. The details of the design of this retrospective cohort study are described elsewhere [32,33]. The initial visit between April 2008 and March 2011 was set as the baseline date. After excluding (i) 176,364 (16.5%) participants with a missing baseline estimated GFR (eGFR), (ii) 110,647 (10.3%) participants with a missing baseline alcohol consumption, (iii) 242,966 (22.7%) participants with missing data on other baseline variables, and (iv) 236,660 (22.1%) participants who had no eGFR measurement during the observational period between their baseline visit and the end of the study in March 2012, we finally included 304,929 (28.5%) participants from 18 prefectures (Hokkaido, Ibaraki, Tochigi, Saitama, Chiba, Niigata, Ishikawa, Fukui, Nagano, Gifu, Osaka, Tokushima, Fukuoka, Saga, Nagasaki, Kumamoto, Miyazaki, and Okinawa) (Figure 1). The study protocol was approved by the Ethics Committees of Fukushima Medical University (No. 2771) and Osaka University Hospital (No. 24086-9).

### 2.2. Measurements

Baseline demographics, physical examinations, and laboratory data at their first visit included age, sex, body mass index (BMI = weight (kg)/height^2^ (m^2^)), mean arterial pressure (diastolic blood pressure + [systolic blood pressure—diastolic blood pressure]/3), hemoglobin A1c, uric acid, high-density lipoprotein (HDL) cholesterol, serum creatinine, eGFR, and dipstick urinary protein. To calculate eGFR, a Japanese equation [34] was used:

194 × age (year)^−0.287^ × serum creatinine (mg/dL)^−1.094^ (× 0.739 if female)

The participants’ baseline drinking and smoking status; current treatments for hypertension, dyslipidemia, and diabetes; and history of cardiovascular disease (CVD) were obtained from standard questionnaires at the baseline visit.

The main exposure of interest in this study was alcohol consumption, which was ascertained by asking the following questions: “How often do you drink alcoholic beverages: (i) every day, (ii) occasionally, or (iii) rarely?” and “How many alcoholic beverages do you drink: (i) <1 drink per day, (ii) 1–2 drinks per day, (iii) 2–3 drinks per day, or (iv) ≥3 drinks per day?”, respectively. One standard drink was defined as 500 mL beer, 180 mL Japanese sake (a traditional Japanese alcoholic beverage), 80 mL shochu (a Japanese liquor), 60 mL whiskey, or 240 mL wine. The ethanol content per one standard drink was calculated to be equivalent to 20 g [35]. Based on these two questions, we classified alcohol consumption into six categories: rare drinkers, occasional drinkers, and daily drinkers with an ethanol intake of ≤19, 20–39, 40–59, and ≥60 g/day.

Participants who answered “Yes” to the “Do you smoke?” question were classified as current smokers. Diagnoses of hypertension, dyslipidemia, and diabetes were made if the participants answered “Yes” to the question, “Are you being treated for hypertension, dyslipidemia, or diabetes?” CVD history was determined according to positive answers to the question, “Have you ever been diagnosed with heart disease and/or stroke?”.

### 2.3. Outcomes

The main outcome of this study was the difference in eGFR slope over time (mL/min/1.73 m^2^/year) between the exposure and reference groups, based on all eGFR measurements at the annual health checkups during the study period between April 2008 and March 2012. The difference in eGFR slope was estimated using linear mixed-effects models, described in Section 2.4 *Statistics* in detail. We also examined the risk for incidence of a ≥30% decline in the eGFR during the observational period. Participants were followed up until March 2012 and censored on the last day of the eGFR measurement at the annual health checkup before the end of March 2012.

### 2.4. Statistics

The baseline clinical characteristics between the included and excluded participants were compared using the χ^2^ test, *t*-test, and Wilcoxon rank sum test, as appropriate. The differences in baseline variables among the alcohol consumption categories were compared using the χ^2^ test, one-way ANOVA, or Kruskal–Wallis test, as appropriate.

The association between alcohol consumption and the eGFR trajectory was assessed using linear mixed-effects models, including all available eGFR values [36,37]. A random intercept was used to account for the variation in baseline eGFR values among participants, and a random slope for time was used to account for the variation in the participants’ eGFR trajectory. The *j*th eGFR of the *i*th participants was estimated using the following equation:

eGFR*_ij_* = ß_0_ + ß_1_Exposre*_i_* + ß_2_Time*_ij_* + ß_3_Time*_ij_* × Expoxure*_i_* + u_0*i*_ + u_2*i*_Time*_ij_* + ε*_ij_*

where ß_1_ represents the estimated difference between the exposure and reference groups, ß_2_ represents the estimated rate of eGFR decline in the reference group, and ß_3_ represents the difference in the eGFR slope between the exposure group and reference groups. The terms *u*_0*i*_ and *u*_2*i*_ represent a random intercept and random slope for time. The estimated differences in eGFR slopes (ß_3_) and their 95% confidence intervals (95% CIs) for each exposure group were reported. To control the potential confounding effects of clinically relevant factors, we used nested linear mixed-effects models, whereby the baseline covariates from each prior model were retained as follows. Model 1 was unadjusted. Model 2 included age (year) as a covariate. Model 3 added urinary protein dipstick values (−, ±, 1+, 2+, and ≥3+). Model 4 added body mass index (kg/m^2^), mean arterial pressure (mmHg), hemoglobin A1c (%), HDL cholesterol (mg/dL), uric acid (mg/dL), and current smoking status. Model 5 added CVD history and current treatments for hypertension, dyslipidemia, and diabetes.

For sensitivity analyses, first, an association between alcohol consumption and the eGFR slope was assessed in 168,347 participants with ≥3 measurements of the eGFR during the observational period; this was after excluding 136,582 participants with 2 measurements of eGFR during the observational period. Second, after excluding 121,431 participants with a CVD history and/or current treatment for hypertension, dyslipidemia, and/or diabetes, we assessed the association between alcohol consumption and the eGFR slope in 183,498 participants without a CVD history or current treatment for hypertension, dyslipidemia, or diabetes, to alleviate the potential impact of sick quitters. Sick quitters who had such comorbidities and, therefore, quit drinking or reduced alcohol consumption [38] might be at a high risk of eGFR decline. The inclusion of sick quitters might lead to a biased estimate of the association between alcohol consumption and the eGFR slope. Third, to clarify the effect of alcohol consumption categorization on the dose-dependent association between alcohol consumption and the eGFR slope, we categorized alcohol consumption into five groups: rare drinkers, occasional drinkers, and daily drinkers with an ethanol intake of ≤19, 20–39, and ≥40 g/day, and calculated the estimated difference in eGFR slopes for each group. Fourth, an association between alcohol consumption and the incidence of a ≥30% decline in the eGFR was assessed by using nested Cox proportional hazards models that were adjusted for clinically relevant factors. Fifth, a propensity score-matched approach was used to compare the eGFR slope and incidence of a ≥30% decline in the eGFRs of rare drinkers and daily drinkers with ≥60 g/day of alcohol consumption with occasional drinkers. Propensity scores, estimated probabilities of being rare drinkers and daily drinkers with ≥60 g/day of alcohol consumption (vs. occasional drinkers), were calculated in separate multivariable-adjusted logistic regression models, including age (year); urinary dipstick protein (−, ±, 1+, 2+, and ≥3+); eGFR (mL/min/1.73 m^2^); body mass index (kg/m^2^); mean arterial pressure (mmHg); hemoglobin A1c (%); HDL cholesterol (mg/dL); uric acid (mg/dL); current smoking; current treatments for hypertension, dyslipidemia, and diabetes; and CVD history as independent variables. After calculating the propensity scores for each patient, each rare drinker and daily drinker with ≥60 g/day of alcohol consumption was matched to occasional drinkers, with the closest propensity score at a ratio of 1:1 and 4:1, respectively, without replacement, using a nearest neighbor matching algorithm with a caliper width of 0.1 standard deviations of the logit of the propensity score [39].

Continuous variables were expressed as the mean ± standard deviation or median (25%–75%), as appropriate, and the categorical variables were expressed as numbers (proportions). The statistical significance was set at *p* < 0.05. In order to perform statistical analyses, we used R software, version 4.1.1 (R Foundation for Statistical Computing, www.r-project.org, accessed on 1 February 2023).

## 3. Results

The baseline clinical characteristics of 125,698 and 325,377 men, who were included in and excluded from the present study, are listed in Table S1. All baseline variables were significantly different between the included and excluded men, except for HDL cholesterol (Table S1). The excluded men were more likely to be current smokers and those with diabetes and CVD history than the included men. Table S2 shows the baseline clinical characteristics of the 179,231 included women and 440,740 excluded women. All variables were statistically different between the included and excluded women, except for BMI.

Table 1 shows the baseline characteristics of 125,698 men, including 38,726 (30.8%) rare drinkers, 32,774 (26.1%) occasional drinkers, and 15,236 (12.1%), 25,819 (20.5%), 10,220 (8.1%), and 2923 (2.3%) daily drinkers with alcohol consumption of ≤19, 20–39, 40–59, and ≥60 g/day, respectively. Daily drinkers with higher alcohol consumption were more likely to be young, current smokers, and hypertensive and had higher levels of uric acid and eGFR, whereas rare drinkers were more prone to dyslipidemia, diabetes, and CVD. The prevalence of proteinuria was comparable among alcohol consumption categories. Contrary to men, most women were rare drinkers, and the prevalence of daily drinkers was very low among women, including 131,484 (73.4%) rare drinkers, 34,874 (19.5%) occasional drinkers, and 7372 (4.1%), 3821 (2.1%), 1152 (0.6%), and 528 (0.4%) daily drinkers with alcohol consumption of ≤19, 20–39, 40–59, and ≥60 g/day, respectively (Table 2). Similar trends in age, smoking status, eGFR, urinary dipstick protein, dyslipidemia, diabetes, and CVD across the alcohol consumption categories were observed among women.

**Figure 2:**
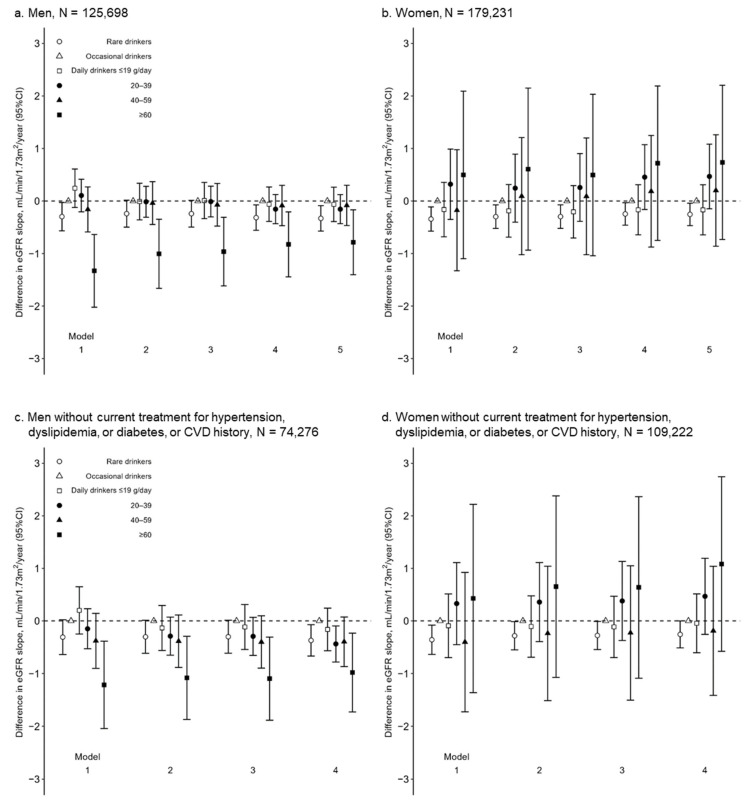
Differences in eGFR slope (ß_3_, mL/min/1.73 m^2^/year) among alcohol consumption categories in multivariable-adjusted linear regression models in 125,698 men (**a**) and 179,231 women (**b**); and 74,276 men (**c**) and 109,222 women (**d**) without current treatment for hypertension, dyslipidemia, or diabetes, or CVD history. CI, confidence interval; CVD, cardiovascular disease; eGFR, estimated glomerular filtration rate. Model 1 unadjusted; Model 2 adjusted for age (year); Model 3 adjusted for age (year) and urinary dipstick protein (−, ±, 1+, 2+, and ≥3+); Model 4 adjusted for covariates in Model 3, body mass index (kg/m^2^), mean arterial pressure (mmHg), hemoglobin A1c (%), high-density lipoprotein cholesterol (mg/dL), uric acid (mg/dL), and current smoking; Model 5 adjusted for covariates in Model 4; current treatment for hypertension, dyslipidemia, and diabetes; and CVD history.

Among 125,698 men, the number of eGFR measurements during the median observational period of 1.9 years (interquartile range 1.1–2.4) was 2, 3, and 4 in 57,589 (45.8%), 47,283 (37.6%), and 20,826 (16.6%) men, respectively (Table S3). An unadjusted model (Model 1) showed that rare drinkers and daily drinkers with alcohol consumption of ≥60 g/day were likely to have a significantly lower eGFR slope than daily drinkers with alcohol consumption of ≤19 g/day (difference in eGFR slope (mL/min/1.73 m^2^/year) of rare drinkers, occasional drinkers, and daily drinkers with alcohol consumptions of ≤19, 20–39, 40–59, and ≥60 g/day: −0.30 [95% CI −0.57, −0.03], 0.00 [reference], 0.24 [−0.12, 0.61], 0.11 [−0.20, 0.41], −0.16 [−0.59, 0.27], and −1.33 [−2.02, −0.64], respectively) (Figure 2a and Table S4). Even after adjusting for clinically relevant factors, daily drinkers with alcohol consumption of ≥60 g/day were associated with significantly lower eGFR slopes than those with alcohol consumption of ≤19 g/day. However, the association between rare drinkers and the eGFR slope was remarkably attenuated (Model 5: −0.33 [−0.57, −0.09], 0.00 [reference], −0.06 [−0.39, 0.26], −0.16 [−0.43, 0.12], −0.08 [−0.47, 0.30], and −0.79 [−1.40, −0.17], respectively) (Figure 2a and Table S4).

The sensitivity analyses verified a U-shape association between alcohol consumption and the eGFR trajectories in men. First, among 68,109 participants with ≥3 measurements of the eGFR during the observational period, a similar dose-dependent association between alcohol consumption and the eGFR slope was observed (Model 5: −0.45 [−0.93, 0.02], 0.00 [reference], −0.16 [−0.80, 0.48], −0.37 [−0.91, 0.17], 0.02 [−0.74, 0.77], and −1.43 [−2.69, −0.17], respectively) (Table S4). Second, after excluding 51,422 men with a CVD history and/or current treatment for hypertension, dyslipidemia, and/or diabetes, an association between alcohol consumption and the eGFR slope was assessed among 74,276 men without a CVD history or current treatment for hypertension, dyslipidemia, or diabetes. Rare drinkers and daily drinkers with ≥60 g/day of alcohol consumption had a significantly higher risk of eGFR decline than occasional drinkers (Model 5: −0.37 [−0.67, −0.07], 0.00 [reference], −0.16 [−0.57, 0.24], −0.44 [−0.78, −0.10], −0.40 [−0.87, 0.07], and −0.98 [−1.73, −0.23], respectively) (Figure 2c and Table S4). Additionally, the association between the alcohol consumption of 20–39 and 40–59 g/day and eGFR decline was more enhanced in the 74,276 men without the mentioned comorbidities than in 125,698 men (daily drinkers with an alcohol consumption of 20–39 and 40–59 g/day in Model 4: −0.44 [−0.78, −0.10] and −0.40 [−0.87, 0.07] in 74,276 men without comorbidities; −0.16 [−0.43, 0.12] and −0.08 [−0.47, 0.30] in 125,698 men, respectively) (Figure 2a,c and Table S4), suggesting that sick quitters might blunt a deleterious effect of alcohol consumption on the eGFR slopes in 125,698 men. Third, if 10,220 and 2923 daily male drinkers with alcohol consumptions of 40–59 and ≥60 g/day were categorized into a single group, this group was no longer associated with the eGFR slope (eGFR slope [mL/min/1.73 m^2^/year] of rare drinkers, occasional drinkers, and daily drinkers with alcohol consumptions of ≤19, 20–39, and ≥40 g/day in Model 5: −0.33 [−0.57, −0.09], 0.00 [reference], −0.06 [−0.39, 0.26], −0.16 [−0.43, 0.12], and −0.23 [−0.57, 0.12], respectively) (Table S5). Fourth, the incidence of a ≥30% decline in the eGFR was observed in 544 (1.4%), 439 (1.3%), 178 (1.2%), 370 (1.4%), 168 (1.6%), and 60 (2.1) men, respectively (Table S6). The multivariable-adjusted Cox proportional hazards model showed a similar association between alcohol consumption and the incidence of a ≥30% decline in the eGFR (1.17 [1.02, 1.34], 1.00 [reference], 0.95 [0.78, 1.14], 1.03 [0.88, 1.20], 1.11 [0.91, 1.35], and 1.21 [0.90, 1.61], respectively), although the association between alcohol consumption of ≥60 g/day and the incidence of a ≥30% decline in the eGFR was not at statistically significant levels, probably because of their small number. Fifth, after calculating the propensity scores of being rare drinkers and daily drinkers with ≥60 g/day of alcohol consumption (vs. occasional drinkers), each rare drinker and daily drinker with ≥60 g/day was matched to occasional drinkers at a ratio of 1:1 and 1:4, respectively. The clinical characteristics of 28,846 rare drinkers and 2901 daily drinkers with ≥60 g/day were clinically comparable with 28,846 and 9825 occasional drinkers, respectively (Table S7). Rare drinkers and daily drinkers with ≥60 g/day had a significantly lower eGFR slope than the occasional drinkers (rare drinkers vs. occasional drinkers, −0.43 [−0.73, −0.13]; daily drinkers with ≥60 g/day vs. occasional drinkers, −0.81 [−1.62, −0.01]) (Table S7). Rare drinkers had a significantly higher risk of a ≥30% decline in the eGFR (hazard ratio, 1.18 [1.01, 1.37]). The hazard ratio of daily drinkers with ≥60 g/day (vs. occasional drinkers) was at the same level as that of rare drinkers but was not at a statistically significant level (1.18 [0.86, 1.62]) because of their small number (Table S7).

Among the 179,231 women, the number of eGFR measurements during the median observational period of 2.0 years (1.1–2.3) were 2, 3, and 4 times in 78,993 (44.1%), 71,063 (39.6%), and 29,175 (16.3%) women, respectively (Table S3). Rare drinkers had significantly lower eGFR slopes than occasional drinkers, whereas daily drinkers did not (Model 5: −0.25 [−0.47, −0.04], 0.00 [reference], −0.17 [−0.64, 0.31], 0.47 [−0.15, 1.08], 0.20 [−0.86, 1.26], and 0.74 [−0.73, 2.20], respectively) (Figure 2b and Table S4). Rare drinkers had significantly lower eGFR slopes than the occasional drinkers among 100,238 women with ≥3 measurements of the eGFR during the observational period and 109,222 women without current treatment for hypertension, dyslipidemia, diabetes, or who had a CVD history (Table S4). The incidence of a ≥30% decline in the eGFR was observed in 2757 (2.1%), 667 (1.9%), 109 (1.5%), 75 (2.0%), 27 (2.3%), and 13 (2.5%) women, respectively (Table S6). The multivariable-adjusted Cox proportional hazard model showed no significant association between alcohol consumption and a ≥30% decline in the eGFR.

## 4. Discussion

This retrospective cohort study clarified a U-shaped association between alcohol consumption and eGFR decline in men. However, in women, daily drinking was not significantly associated with the eGFR trajectory, while rare drinkers were significantly more vulnerable to eGFR decline than occasional drinkers in women, like men. A large sample size enabled us to perform a statistically meaningful analysis of the critical impact of heavy drinking (≥60 g/day) on eGFR decline in men. However, the low prevalence of daily drinking in women hinders the assessment of their clinical impact on the eGFR trajectory.

Although multiple cohort studies have assessed the clinical impact of alcohol consumption on eGFR trajectory, most studies have defined the largest alcohol consumption as >10–30 g/day [24,25,26,27], partly because of their limited sample sizes. The results of this study strongly suggest that these previous studies might have underestimated the deleterious effects of heavy drinking (Table S5). Few studies have assessed the clinical impact of alcohol consumption of >40 g/day on eGFR trajectory. The Kansai Healthcare Study, including 9112 male workers in a single company in Japan, reported an inverse J-shaped association between alcohol consumption and the incidence of a low eGFR of <60 mL/min/1.73 m^2^ during a median observational period of 10.5 years (multivariable-adjusted hazard ratio [95% confidence interval] of non-drinkers and current drinkers with an alcohol consumption of ≤23.0, 23.1–46.0, 46.1–69.0, and ≥69.1 g/day: 1.00 [reference], 0.89 [0.76, 1.04], 0.65 [0.55, 0.77], 0.77 [0.61, 0.77], and 0.76 [0.43, 1.37], respectively) [28]. However, among 102 drinkers with an alcohol consumption ≥69.1 g/day, only 12 drinkers developed a low eGFR, suggesting that the incidence of having a low eGFR was too small to estimate the risk of low eGFR precisely. Another small cohort study, the Italian Longitudinal Study on Aging (ILSA), assessed a dose-dependent association between alcohol consumption (abstainers, former drinkers, and current drinkers with alcohol consumptions of ≤12, 13–24, 25–47, and ≥48 g/day) and the incidence of a low eGFR of <60 mL/min/1.73 m^2^ among 886 older men and 653 older women, in whom the incidence of low eGFRs was observed in 91 participants during the mean observational period of 3.5 years [31]. This study, with low statistical power, showed that only former male drinkers were significantly associated with an incidence of a low eGFR (multivariable-adjusted odds ratio [95% confidence interval] of former drinkers vs. abstainers: 0.20 [0.05, 0.87]), and current drinkers were not associated with the incidence of low eGFRs in either men or women. The remarkably large sample size of this study enabled us to statistically analyze the clinical impact of heavy drinking on eGFRs in men. However, this study lacked the power to assess the clinical impact of heavy drinking on the eGFR trajectory in women with a very low prevalence of alcohol consumption of ≥60 g/day. A larger number of female heavy drinkers was essential to clarify the association between heavy alcohol consumption and the eGFR trajectory in women.

The exclusion of men with a CVD history and/or current treatment for hypertension, dyslipidemia, and/or diabetes clarified a negative linear association between alcohol consumption and the eGFR trajectory in male daily drinkers (Figure 2c). Current drinkers are likely to decrease alcohol consumption and quit drinking after the incidence of cardiometabolic diseases, including diabetes and heart diseases [38,40]. Patients with these cardiometabolic diseases are at high risk for CKD [26,41]. Herein, sick quitters who reduced alcohol consumption after the incidence of cardiometabolic diseases might be classified into lower alcohol consumption categories than those before the incidence of the cardiometabolic disease, possibly leading to an attenuation of the beneficial effect of mild alcohol consumption on eGFR (Figure 2a). The exclusion of participants with these cardiometabolic diseases might alleviate the sick-quitter effect, clarifying the dose-dependent association between alcohol consumption and the eGFR slope (Figure 2c).

Aside from the sick-quitter effect, an anti-inflammatory effect of mild alcohol consumption might contribute to a significantly lower risk for eGFR decline in occasional drinkers than that of rare drinkers. Mild drinkers have lower levels of inflammatory markers than non-drinkers, including C-reactive protein [42]. These inflammatory markers are risk factors for GFR decline [43,44]. Thus, occasional drinkers with lower inflammatory levels might be less vulnerable to eGFR decline than rare drinkers in this study.

This study had several limitations. First, alcohol consumption was self-reported in this study, possibly leading to a misclassification bias. Measurement of the biomarkers of alcohol consumption, including urinary ethyl glucuronide [45], is desirable to confirm the validity of this study. Second, the median observational period of 1.9 [1.1–2.3] years was short, and information on ESKD was unavailable in this study. Cohort studies with longer observational periods are necessary to estimate the clinical impact of alcohol consumption on long-term kidney function and the incidence of ESKD. Third, information regarding the type of alcoholic beverages was not available in this study. A Chinese cohort study reported that liquor consumption was associated with a significantly lower risk of ESKD incidence, whereas non-liquor consumption was not [22], suggesting that the beneficial effect of alcohol consumption might be dependent on specific types of alcoholic beverages. Fourth, unmeasured confounding factors may have affected the association between alcohol consumption and the eGFR slope. One potential confounding factor may be salt intake. A recent large cross-sectional study, including 10,762 Japanese participants, reported that higher alcohol consumption is associated with higher salt intake [46]. Because high salt intake is a risk factor for a decline in GFR [47], the association between alcohol consumption and the eGFR slope should be strengthened after an adjustment for the confounding factors associated with salt intake.

## 5. Conclusions

In conclusion, this retrospective cohort study showed that the men who were rare drinkers and current drinkers with a heavy alcohol consumption of ≥60 g/day were at a higher risk of a decline in eGFR than those of men who were occasional drinkers, suggesting that alcohol consumption was associated with the eGFR trajectory in a U-shaped fashion in men. Because of the low prevalence of high alcohol consumption among women, the association between high alcohol consumption and the eGFR trajectory remains unknown in women in this study. Unmeasured confounding factors might affect the association between alcohol consumption and the eGFR decline observed in this study. A well-designed cohort study with a long follow-up period is necessary to assess the long-term clinical impact of high alcohol consumption on the eGFR trajectory.

## Figures and Tables

**Table 1:** Baseline characteristics of 125,698 men stratified by alcohol consumption.

	Rare	Occasional	Daily			
			≤19 g/day	20–39	40–59	≥60
N	38,726	32,774	15,236	25,819	10,220	2923
Age, year	66 (59–70)	65 (58–69)	67 (62–70)	66 (60–69)	64 (57–68)	60 (52–65)
Smokers, N (%)	9166 (23.7)	7275 (22.2)	3558 (23.4)	7722 (29.9)	4007 (39.2)	1290 (44.1)
BMI, kg/m^2^	23.9 ± 3.2	24.1 ± 3.1	23.5 ± 2.8	23.6 ± 2.8	23.7 ± 3.0	23.9 ± 3.1
SBP, mmHg	128 ± 17	130 ± 17	131 ± 17	134 ± 17	135 ± 17	135 ± 18
DBP, mmHg	77 ± 11	78 ± 11	78 ± 10	80 ± 11	81 ± 11	82 ± 11
MAP, mmHg	94 ± 12	96 ± 12	96 ± 11	98 ± 12	99 ± 12	100 ± 12
HDL cholesterol, mg/dL	52 ± 13	56 ± 14	59 ± 15	61 ± 16	63 ± 17	64 ± 18
Hemoglobin A1c, %	5.5 ± 0.8	5.4 ± 0.8	5.4 ± 0.7	5.3 ± 0.7	5.3 ± 0.7	5.3 ± 0.8
Uric acid, mg/dL	5.8 ± 1.3	6.0 ± 1.3	6.0 ± 1.3	6.1 ± 1.3	6.3 ± 1.4	6.5 ± 1.4
eGFR, mL/min/1.73 m^2^	72 (63–82)	73 (64–84)	73 (64–84)	74 (65–85)	76 (66–87)	78 (69–89)
≥90, N (%)	3841 (9.9)	3847 (11.7)	1533 (10.1)	3377 (13.1)	1923 (18.8)	695 (23.8)
60–89	26,328 (68.0)	23,037 (70.3)	10,688 (70.1)	18,553 (71.9)	7100 (69.5)	1957 (67.0)
45–59	7495 (19.4)	5302 (16.2)	2723 (17.9)	3522 (13.6)	1104 (10.8)	247 (8.5)
<45	1062 (2.7)	588 (1.8)	292 (1.9)	367 (1.4)	93 (0.9)	24 (0.8)
Dipstick UP, −, N (%)	31,949 (82.5)	26,978 (82.3)	12,919 (84.8)	21,553 (83.5)	8287 (81.1)	2309 (79.0)
±	3756 (9.7)	3353 (10.2)	1337 (8.8)	2474 (9.6)	1111 (10.9)	343 (11.7)
1+	1955 (5.0)	1617 (4.9)	650 (4.3)	1232 (4.8)	571 (5.6)	178 (6.1)
2+	815 (2.1)	619 (1.9)	253 (1.7)	424 (1.6)	204 (2.0)	75 (2.6)
≥3+	251 (0.6)	207 (0.6)	77 (0.5)	136 (0.5)	47 (0.5)	18 (0.6)
Hypertension, N (%)	10,872 (28.1)	10,030 (30.6)	4959 (32.5)	9115 (35.3)	3540 (34.6)	873 (29.9)
Dyslipidemia, N (%)	4486 (11.6)	3503 (10.7)	1555 (10.2)	2376 (9.2)	810 (7.9)	199 (6.8)
Diabetes, N (%)	3099 (8.0)	2397 (7.3)	919 (6.0)	1432 (5.5)	524 (5.1)	177 (6.1)
CVD history, N (%)	5019 (13.0)	3475 (10.6)	1726 (11.3)	2580 (10.0)	858 (8.4)	167 (5.7)

Mean ± SD; median (25%–75%), BMI, body mass index; CVD, cardiovascular disease; DBP, diastolic blood pressure; eGFR, estimated glomerular filtration rate; HDL, high-density lipoprotein; MAP, mean arterial pressure; SBP, systolic blood pressure; UP, urinary protein. *p* < 0.05 for all variables.

**Table 2:** Baseline characteristics of 179,231 women stratified by alcohol consumption.

	Rare	Occasional	Daily			
			≤19 g/day	20–39	40–59	≥60
N	131,484	34,874	7372	3821	1152	528
Age, year	65 (60–69)	64 (58–68)	64 (58–68)	60 (53–65)	56 (49–62)	55 (46–61)
Smokers, N (%)	5650 (4.3)	2528 (7.2)	694 (9.4)	886 (23.2)	423 (36.7)	222 (42.0)
BMI, kg/m^2^	23.0 ± 3.5	22.7 ± 3.2	22.0 ± 2.9	22.2 ± 3.2	22.1 ± 3.3	22.8 ± 3.4
SBP, mmHg	128 ± 18	127 ± 18	127 ± 18	128 ± 18	127 ± 18	128 ± 18
DBP, mmHg	75 ± 10	75 ± 11	75 ± 11	76 ± 11	77 ± 11	77 ± 11
MAP, mmHg	93 ± 12	92 ± 12	92 ± 12	94 ± 12	93 ± 12	94 ± 13
HDL cholesterol, mg/dL	64 ± 15	67 ± 16	72 ± 17	75 ± 18	77 ± 19	76 ± 20
Hemoglobin A1c, %	5.3 ± 0.6	5.3 ± 0.5	5.2 ± 0.5	5.1 ± 0.5	5.1 ± 0.6	5.1 ± 0.6
Uric acid, mg/dL	4.6 ± 1.1	4.7 ± 1.0	4.7 ± 1.0	5.0 ± 1.2	5.2 ± 1.2	5.4 ± 1.3
eGFR, mL/min/1.73 m^2^	74 (64–83)	75 (64–86)	75 (64–85)	77 (67–91)	80 (72–94)	82 (72–96)
≥90, N (%)	26,443 (20.1)	7678 (22.0)	1563 (21.2)	997 (26.1)	382 (33.2)	189 (35.8)
60–89	87,823 (66.8)	23,534 (67.5)	5068 (68.7)	2541 (66.5)	719 (62.4)	308 (58.3)
45–59	15,497 (11.8)	3402 (9.8)	693 (9.4)	264 (6.9)	49 (4.3)	29 (5.5)
<45	1721 (1.3)	260 (0.7)	48 (0.7)	19 (0.5)	2 (0.2)	2 (0.4)
Dipstick UP, −, N (%)	117,136 (89.1)	31,244 (89.6)	6723 (91.2)	3424 (89.6)	999 (86.7)	431 (81.6)
±	9246 (7.0)	2465 (7.1)	443 (6.0)	253 (6.6)	108 (9.4)	61 (11.6)
1+	3705 (2.8)	877 (2.5)	161 (2.2)	109 (2.9)	38 (3.3)	22 (4.2)
2+	1112 (0.8)	229 (0.7)	35 (0.5)	29 (0.8)	5 (0.4)	13 (2.5)
≥3+	285 (0.2)	59 (0.2)	10 (0.1)	6 (0.2)	2 (0.2)	1 (0.2)
Hypertension, N (%)	36,581 (27.8)	8206 (23.5)	1642 (22.3)	969 (25.4)	259 (22.5)	126 (23.9)
Dyslipidemia, N (%)	25,628 (19.5)	5578 (16.0)	977 (13.3)	371 (9.7)	91 (7.9)	33 (6.3)
Diabetes, N (%)	5677 (4.3)	848 (2.4)	137 (1.9)	65 (1.7)	16 (1.4)	11 (2.1)
CVD history, N (%)	9316 (7.1)	2085 (6.0)	439 (6.0)	227 (5.9)	55 (4.8)	32 (6.1)

Mean ± SD; median (25%–75%). BMI, body mass index; CVD, cardiovascular disease; DBP, diastolic blood pressure; eGFR, estimated glomerular filtration rate; HDL, high-density lipoprotein; MAP, mean arterial pressure; SBP, systolic blood pressure; UP, urinary protein. *p* < 0.05 for all variables.

## Data Availability

The data supporting the findings of this study are available from the corresponding author upon reasonable request.

## Supplementary Materials

The following supporting information can be downloaded at: https://www.mdpi.com/article/10.3390/nu15061540/s1, Table S1: Baseline characteristics of included and excluded men; Table S2: Baseline characteristics of included and excluded women; Table S3: Observational period and numbers of eGFR measurements; Table S4: Alcohol consumption of six categories and eGFR slope in 125,698 men and 179,231 women; Table S5: Alcohol consumption of five categories and eGFR slope in 125,698 men and 179,231 women; Table S6: Alcohol consumption and eGFR slope in 74,276 men and 109,222 women without current treatment for hypertension, dyslipidemia, or diabetes, or history of cardiovascular disease; Table S7: Baseline characteristics and outcomes of rare vs. occasional male drinkers (1:1) and rare vs. daily male drinkers with alcohol consumption of ≥60 g/day (4:1), matched by propensity score.
