# Supplementary material for: Alcohol Consumption and a Decline in Glomerular Filtration Rate: The Japan Specific Health Checkups Study
Source: Nutrients. 2023 Mar 22;15(6):1540. doi: 10.3390/nu15061540 (PMC10058733; doi:10.3390/nu15061540)
Supplement: Supplementary file 1 [file nutrients-15-01540-s001.zip › nutrients-2240085-supplementary.pdf]

nutrients

Article

Alcohol Consumption and a decline in glomerular filtration rate: the Japan Specific Health Checkups study

Yoshiki Kimura, Ryohei Yamamoto, Maki Shinzawa, Katsunori Aoki, Ryohei Tomi, Shingo Ozaki, Ryuichi Yoshimura, Akihiro Shimomura, Hirotsugu Iwatani, Yoshitaka Isaka, Kunitoshi Iseki, Kazuhiko Tsuruya, Shouichi Fujimoto, Ichiei Narita, Tsuneo Konta, Masahide Kondo, Masato Kasahara, Yugo Shibagaki, Koichi Asahi, Tsuyoshi Watanabe, Kunihiro Yamagata, and Toshiki Moriyama

Supplementary materials

Table S1. Baseline characteristics of included and excluded men.
Table S2. Baseline characteristics of included and excluded women.
Table S3. Observational period and numbers of eGFR measurements.
Table S4. Difference in eGFR slope among alcohol consumption categories.
Table S5. Difference in eGFR slope among five categories of alcohol consumption (vs. occasional drinkers, mL/min/1.73 m^2/year) in 125,698 men and 179,231 women.
Table S6. Alcohol consumption and incidence of ≥ 30% decline in eGFR in 125,698 men and 179,231 women.
Table S7. Baseline characteristics and outcomes of rare vs. occasional male drinkers (1:1) and rare vs. daily male drinkers with alcohol consumption of ≥ 60 g/day (4:1) matched by propensity score.

Table S1. Baseline characteristics of included and excluded men.

	Included	Excluded	Missing, N (%)
Number	125,698	325,377	
Age, year	66 (59–69)	65 (57–69)	0 (0.0)
BMI, kg/m^2	23.8 ± 3.0	23.8 ± 3.1	3,514 (1.1)
Current smokers, N (%)	33,018 (26.3)	95,469 (30.6)	13,125 (4.0)
Drinking status			54,327 (16.7)
Rare drinkers, N (%)	38,726 (30.8)	83,238 (30.7)	
Occasional drinkers	32,774 (26.1)	67,479 (24.9)	
Daily drinkers ≤ 19 g/day	15,236 (12.1)	30,293 (11.2)	
20–39	25,819 (20.5)	55,885 (20.6)	
40–59	10,220 (8.1)	26,530 (9.8)	
≥ 60	2,923 (2.3)	7,625 (2.8)	
SBP, mmHg	131 ± 17	132 ± 18	36,173 (11.1)
DBP, mmHg	78 ± 11	79 ± 11	36,266 (11.1)
MAP, mmHg	96 ± 12	97 ± 12	36,281 (11.2)
HDL cholesterol, mg/dL	57 ± 15	57 ± 15	245 (0.1)
Hemoglobin A1c, %	5.4 ± 0.8	5.4 ± 0.9	17,574 (5.4)
Uric acid, mg/dL	6.0 ± 1.3	6.0 ± 1.4	115,961 (35.6)
eGFR, ml/min/1.73 m^2	73 (64–84)	74 (65–85)	74,109 (22.8)
≥ 90, N (%)	15,216 (12.1)	39502 (15.7)	
60–89	87,663 (69.7)	171,168 (68.1)	
45–59	20,393 (16.2)	35,233 (14.0)	
< 45	2,426 (1.9)	5,365 (2.1)	
Dipstick UP, -, N (%)	103,995 (82.7)	267,903 (83.3)	3,722 (1.1)
±	12,374 (9.8)	28,497 (8.9)	
1+	6,203 (4.9)	16,620 (5.2)	
2+	2,390 (1.9)	6,414 (2.0)	
≥ 3+	736 (0.6)	2,221 (0.7)	
Hypertension, N (%)	39,389 (31.3)	100,990 (31.8)	7,570 (2.3)
Dyslipidemia, N (%)	12,929 (10.3)	34,364 (10.8)	8,182 (2.5)
Diabetes, N (%)	8,548 (6.8)	24,901 (7.9)	8,350 (2.6)
CVD history, N (%)	13,825 (11.0)	32,727 (12.0)	51,814 (15.9)

Mean ± SD; Median (25%–75%)

BMI, body mass index; CVD, cardiovascular disease; DBP, diastolic blood pressure; eGFR, estimated glomerular filtration rate; HDL, high density lipoprotein; MAP, mean arterial pressure; SBP, systolic blood pressure; UP, urinary protein.

Table S2. Baseline characteristics of included and excluded women.

		Included	Excluded	Missing, N (%)
Number		179,231	440,740	
Age, year		65 [59–69]	64 [58–69]	0 (0.0)
BMI, kg/m^2		22.9 ± 3.4	22.9 ± 3.5	4,556 (1.0)
Current smokers, N (%)		10,403 (5.8)	33,098 (7.9)	19,665 (4.5)
Drinking status				67,590 (15.3)
Rare drinkers, N (%)		131,484 (73.4)	263,452 (70.6)	
Occasional drinkers		34,874 (19.5)	78,893 (21.1)	
Daily drinkers	≤ 19 g/day	7,372 (4.1)	15,676 (4.2)	
	20–39	3,821 (2.1)	10,231 (2.7)	
	40–59	1,152 (0.6)	3,473 (0.9)	
	≥ 60	528 (0.3)	1,425 (0.4)	
SBP, mmHg		128 ± 18	128 ± 18	53,966 (12.2)
DBP, mmHg		75 ± 10	75 ± 11	54,133 (12.3)
MAP, mmHg		92 ± 12	93 ± 12	54,155 (12.3)
HDL cholesterol, mg/dL		65 ± 16	66 ± 16	347 (0.1)
Hemoglobin A1c, %		5.3 ± 0.6	5.3 ± 0.6	23,159 (5.3)
Uric acid, mg/dL		4.6 ± 1.1	4.6 ± 1.1	162,546 (36.9)
eGFR, ml/min/1.73 m^2		75 (64–84)	75 (64–89)	101,735 (23.1)
≥ 90, N (%)		37,252 (20.8)	76,590 (22.6)	
60–89		1119,993 (66.9)	222,241 (65.6)	
45–59		19,934 (11.1)	35,457 (10.5)	
< 45		2,052 (1.1)	4,717 (1.4)	
Dipstick UP,	-, N (%)	159,957 (89.2)	391,047 (89.9)	5,543 (1.3)
	±	12,576 (7.0)	27,489 (6.3)	
	1+	4,912 (2.7)	12,171 (2.8)	
	2+	1,423 (0.8)	3,494 (0.8)	
	≥ 3+	363 (0.2)	996 (0.2)	
Hypertension, N (%)		47,783 (26.7)	119,807 (27.9)	11,408 (2.6)
Dyslipidemia, N (%)		32,678 (18.2)	79,342 (18.5)	11,281 (2.6)
Diabetes, N (%)		6,754 (3.8)	18,261 (4.3)	12,533 (2.8)
CVD history, N (%)		12,154 (6.8)	28,178 (7.6)	67,761 (15.4)

Mean ± SD; Median (25%–75%)

BMI, body mass index; CVD, cardiovascular disease; DBP, diastolic blood pressure; eGFR, estimated glomerular filtration rate; HDL, high density lipoprotein; MAP, mean arterial pressure; SBP, systolic blood pressure; UP, urinary protein.

$P < 0.05$ for all variables except body mass index.

Table S3. Observational period and numbers of eGFR measurements .

	Rare	Occasional	Daily			
			≤ 19 g/day	20–39	40–59	≥ 60
**Men, N = 125,698**						
Observational period, year	1.9 (1.1–2.4)	2.0 (1.1–2.5)	1.9 (1.1–2.3)	1.9 (1.1–2.2)	1.9 (1.1–2.2)	2.0 (1.1–2.6)
Measurements of eGFR						
2 times, N (%)	18,014 (46.5)	14,752 (45.0)	6,948 (45.6)	11,688 (45.3)	4,819 (47.2)	1,368 (46.8)
3	13,875 (35.8)	11,879 (36.2)	6,060 (39.8)	10,433 (40.4)	3,996 (39.1)	1,040 (35.6)
4	6,837 (17.7)	6,143 (18.7)	2,228 (14.6)	3,698 (14.3)	1,405 (13.7)	515 (17.6)
**Women, N = 179,231**						
Observational period, year	2.0 (1.1–2.4)	1.9 (1.1–2.2)	1.9 (1.1–2.1)	1.9 (1.1–2.2)	1.9 (1.1–2.2)	1.9 (1.0–2.3)
Measurements of eGFR						
2 times, N (%)	56,755 (43.2)	16,050 (46.0)	3,409 (46.2)	1,889 (49.4)	602 (52.3)	288 (54.5)
3	51,334 (39.0)	14,401 (41.3)	3,199 (43.4)	1,526 (39.9)	427 (37.1)	176 (33.3)
4	23,395 (17.8)	4,423 (12.7)	764 (10.4)	406 (10.6)	123 (10.7)	64 (12.1)

Median (25%–75%)
eGFR, estimated glomerular filtration rate.

Table S4. Difference in eGFR slope among alcohol consumption categories

	Difference in eGFR slope, mL/min/1.73 m²/year (95% CI)					
	Rare	Occasional	Daily, ≤ 19 g/day	20–39	40–59	≥ 60
**All men, N = 125,698**						
N	38,726	32,774	15,236	25,819	10,220	2,923
Model 1	-0.30 (-0.57, -0.03)*	0.00 (reference)	0.24 (-0.12, 0.61)	0.11 (-0.20, 0.41)	-0.16 (-0.59, 0.27)	-1.33 (-2.02, -0.64)*
Model 2	-0.24 (-0.50, 0.02)	0.00 (reference)	-0.01 (-0.36, 0.34)	-0.01 (-0.31, 0.28)	-0.04 (-0.45, 0.37)	-1.01 (-1.66, -0.35)*
Model 3	-0.24 (-0.50, 0.01)	0.00 (reference)	0.01 (-0.34, 0.36)	-0.01 (-0.30, 0.28)	-0.07 (-0.48, 0.33)	-0.96 (-1.62, -0.31)*
Model 4	-0.32 (-0.56, -0.07)*	0.00 (reference)	-0.06 (-0.39, 0.27)	-0.16 (-0.43, 0.12)	-0.09 (-0.47, 0.30)	-0.82 (-1.44, -0.21)*
Model 5	-0.33 (-0.57, -0.09)*	0.00 (reference)	-0.06 (-0.39, 0.26)	-0.16 (-0.43, 0.12)	-0.08 (-0.47, 0.30)	-0.79 (-1.40, -0.17)*
**Men with ≥ 3 measurements of eGFR during observational period, N = 68,109**						
N	20,712	18,022	8,288	14,131	5,401	1,555
Model 1	-0.67 (-1.20, -0.14)*	0.00 (reference)	0.16 (-0.56, 0.87)	0.02 (-0.58, 0.62)	-0.03 (-0.87, 0.82)	-1.90 (-3.31, -0.50)*
Model 2	-0.31 (-0.81, 0.20)	0.00 (reference)	-0.05 (-0.73, 0.63)	-0.28 (-0.85, 0.29)	0.03 (-0.77, 0.83)	-1.55 (-2.90, -0.21)*
Model 3	-0.31 (-0.81, 0.19)	0.00 (reference)	-0.04 (-0.72, 0.64)	-0.27 (-0.84, 0.29)	-0.04 (-0.84, 0.76)	-1.57 (-2.90, -0.23)*
Model 4	-0.45 (-0.93, 0.02)	0.00 (reference)	-0.18 (-0.82, 0.47)	-0.40 (-0.94, 0.14)	0.00 (-0.76, 0.76)	-1.52 (-2.79, -0.25)*
Model 5	-0.45 (-0.93, 0.02)	0.00 (reference)	-0.16 (-0.80, 0.48)	-0.37 (-0.91, 0.17)	0.02 (-0.74, 0.77)	-1.43 (-2.69, -0.17)*
**Men without current treatment for hypertension, dyslipidemia, or diabetes, or CVD history, N = 74,276**						
N	23,226	19,479	8,872	14,773	6,047	1,879
Model 1	-0.31 (-0.64, 0.02)	0.00 (reference)	0.20 (-0.25, 0.65)	-0.15 (-0.53, 0.23)	-0.38 (-0.90, 0.14)	-1.21 (-2.04, -0.39)*
Model 2	-0.30 (-0.62, 0.01)	0.00 (reference)	-0.13 (-0.56, 0.29)	-0.29 (-0.65, 0.07)	-0.39 (-0.88, 0.11)	-1.08 (-1.87, -0.29)*
Model 3	-0.30 (-0.62, 0.01)	0.00 (reference)	-0.12 (-0.54, 0.31)	-0.29 (-0.66, 0.07)	-0.40 (-0.90, 0.10)	-1.10 (-1.88, -0.31)*
Model 4	-0.37 (-0.67, -0.07)*	0.00 (reference)	-0.16 (-0.57, 0.24)	-0.44 (-0.78, -0.10)*	-0.40 (-0.87, 0.07)	-0.98 (-1.73, -0.23)*
**All women, N = 179,231**						
N	131,484	34,874	7,372	3,821	1,152	528
Model 1	-0.34 (-0.57, -0.11)*	0.00 (reference)	-0.16 (-0.68, 0.36)	0.32 (-0.35, 0.99)	-0.18 (-1.33, 0.98)	0.50 (-1.10, 2.09)
Model 2	-0.30 (-0.52, -0.07)*	0.00 (reference)	-0.19 (-0.69, 0.31)	0.24 (-0.40, 0.89)	0.09 (-1.02, 1.21)	0.61 (-0.93, 2.15)
Model 3	-0.30 (-0.52, -0.07)*	0.00 (reference)	-0.21 (-0.70, 0.29)	0.26 (-0.39, 0.90)	0.09 (-1.02, 1.20)	0.49 (-1.04, 2.03)
Model 4	-0.25 (-0.46, -0.03)*	0.00 (reference)	-0.17 (-0.64, 0.31)	0.45 (-0.16, 1.07)	0.19 (-0.88, 1.25)	0.72 (-0.75, 2.19)
Model 5	-0.25 (-0.47, -0.04)*	0.00 (reference)	-0.17 (-0.64, 0.31)	0.47 (-0.15, 1.08)	0.20 (-0.86, 1.26)	0.74 (-0.73, 2.20)
**Women with ≥ 3 measurements of eGFR during observational period, N = 100,238**						
N	74,729	18,824	3,963	1,932	550	240
Model 1	-0.93 (-1.38, -0.48)*	0.00 (reference)	-0.04 (-1.05, 0.98)	0.36 (-0.98, 1.69)	-0.81 (-3.22, 1.60)	1.98 (-1.24, 5.21)
Model 2	-0.47 (-0.90, -0.03)*	0.00 (reference)	-0.14 (-1.13, 0.84)	0.08 (-1.20, 1.37)	-0.31 (-2.64, 2.02)	2.44 (-0.68, 5.55)
Model 3	-0.48 (-0.91, -0.05)*	0.00 (reference)	-0.20 (-1.18, 0.78)	0.11 (-1.17, 1.39)	-0.40 (-2.72, 1.93)	2.23 (-0.87, 5.33)
Model 4	-0.53 (-0.95, -0.12)*	0.00 (reference)	-0.33 (-1.27, 0.60)	0.50 (-0.73, 1.73)	-0.10 (-2.32, 2.12)	2.29 (-0.69, 5.26)
Model 5	-0.55 (-0.97, -0.14)*	0.00 (reference)	-0.35 (-1.29, 0.59)	0.48 (-0.75, 1.70)	-0.11 (-2.33, 2.11)	2.28 (-0.69, 5.25)
**Women without current treatment for hypertension, dyslipidemia, or diabetes, or CVD history, N = 109,222**						
N	77,672	22,753	5,004	2,583	835	375
Model 1	-0.36 (-0.64, -0.08)*	0.00 (reference)	-0.09 (-0.70, 0.51)	0.33 (-0.45, 1.11)	-0.40 (-1.73, 0.92)	0.43 (-1.36, 2.22)
Model 2	-0.28 (-0.55, -0.02)*	0.00 (reference)	-0.11 (-0.69, 0.48)	0.36 (-0.39, 1.11)	-0.24 (-1.51, 1.04)	0.65 (-1.07, 2.38)
Model 3	-0.28 (-0.55, -0.01)*	0.00 (reference)	-0.11 (-0.70, 0.47)	0.38 (-0.37, 1.13)	-0.23 (-1.50, 1.05)	0.64 (-1.09, 2.36)
Model 4	-0.26 (-0.52, -0.00)*	0.00 (reference)	-0.05 (-0.61, 0.51)	0.47 (-0.26, 1.19)	-0.19 (-1.42, 1.04)	1.08 (-0.58, 2.74)

CI, confidence interval; CVD, cardiovascular disease; eGFR, estimated glomerular filtration rate.
*P for interaction between alcohol consumption category and time < 0.05
Model 1, unadjusted.
Model 2, adjusted for age (year).
Model 3, adjusted for age (year) and dipstick urinary protein (-, ±, 1+, 2+, and ≥ 3+).

Model 4, adjusted for covariates in Model 3 and body mass index (kg/m^2), mean arterial pressure (mmHg), hemoglobin A1c (%), high density lipoprotein cholesterol (mg/dL), uric acid (mg/dL), and current smoking.
Model 5, adjusted for covariates in Model 4 and current treatment for hypertension, dyslipidemia and diabetes, and history of cardiovascular disease.

Table S5. Difference in eGFR slope among five categories of alcohol consumption (vs. occasional drinkers, mL/min/1.73 m^2/year) in 125,698 men and 179,231 women

	Difference in eGFR slope, mL/min/1.73 m^2/year (95% CI)				
	Rare	Occasional	Daily, ≤ 19 g/day	20–39	≥ 40
**Men, N = 125,698**					
N	38,726	32,774	15,236	25,819	13,123
Model 1	-0.30 (-0.57, -0.03)*	0.00 (reference)	0.24 (-0.12, 0.61)	0.10 (-0.20, 0.41)	-0.38 (-0.76, 0.01)
Model 2	-0.24 (-0.50, 0.02)	0.00 (reference)	-0.01 (-0.36, 0.34)	-0.01 (-0.31, 0.28)	-0.26 (-0.63, 0.10)
Model 3	-0.24 (-0.50, 0.01)	0.00 (reference)	0.01 (-0.34, 0.35)	-0.01 (-0.30, 0.28)	-0.27 (-0.64, 0.09)
Model 4	-0.32 (-0.56, -0.07)*	0.00 (reference)	-0.06 (-0.39, 0.27)	-0.16 (-0.43, 0.12)	-0.24 (-0.58, 0.11)
Model 5	-0.33 (-0.57, -0.09)*	0.00 (reference)	-0.06 (-0.39, 0.26)	-0.16 (-0.43, 0.12)	-0.23 (-0.57, 0.12)
**Women, N = 179,231**					
N	131,484	34,874	7,372	3,821	1,680
Model 1	-0.34 (-0.57, -0.11)*	0.00 (reference)	-0.16 (-0.68, 0.36)	0.32 (-0.35, 0.99)	0.07 (-0.88, 1.01)
Model 2	-0.30 (-0.52, -0.07)*	0.00 (reference)	-0.19 (-0.69, 0.31)	0.24 (-0.40, 0.89)	0.27 (-0.64, 1.18)
Model 3	-0.30 (-0.52, -0.07)*	0.00 (reference)	-0.21 (-0.70, 0.29)	0.26 (-0.39, 0.90)	0.23 (-0.68, 1.14)
Model 4	-0.25 (-0.46, -0.03)*	0.00 (reference)	-0.17 (-0.64, 0.31)	0.45 (-0.16, 1.07)	0.38 (-0.49, 1.25)
Model 5	-0.25 (-0.47, -0.04)*	0.00 (reference)	-0.17 (-0.64, 0.31)	0.47 (-0.15, 1.08)	0.40 (-0.47, 1.27)

CI, confidence interval; eGFR, estimated glomerular filtration rate.

*P for interaction between alcohol consumption category and time < 0.05

Model 1, unadjusted.

Model 2, adjusted for age (year).

Model 3, adjusted for age (year) and dipstick urinary protein (-, ±, 1+, 2+, and ≥ 3+).

Model 4, adjusted for covariates in Model 3 and body mass index (kg/m^2), mean arterial pressure (mmHg), hemoglobin A1c (%), high density lipoprotein cholesterol (mg/dL), uric acid (mg/dL), and current smoking.

Model 5, adjusted for covariates in Model 4 and current treatment for hypertension, dyslipidemia and diabetes, and history of cardiovascular disease.

nutrients MDPI

Table S6. Alcohol consumption and incidence of ≥ 30% decline in eGFR in 125,698 men and 179,231 women.

		Rare	Occasional	Daily ≤ 19 g/day	20–39	40–59	≥ 60
Men, N		38,726	32,774	15,236	25,819	10,220	2,923
Observational period, yr		2.0 (1.3–2.9)	2.0 (1.4–2.9)	2.0 (1.3–2.7)	2.0 (1.3–2.6)	2.0 (1.2–2.6)	2.0 (1.4–2.9)
eGFR decline ≥ 30%, N (%)		544 (1.4)	439 (1.3)	178 (1.2)	370 (1.4)	168 (1.6)	60 (2.1)
HR (95% CI),	model 1	1.12 (0.98, 1.28)	1.00 (reference)	0.96 (0.79, 1.16)	1.19 (1.03, 1.38)*	1.43 (1.18, 1.73)*	1.57 (1.18, 2.09)*
	model 2	1.09 (0.95, 1.25)	1.00 (reference)	0.91 (0.75, 1.10)	1.16 (1.00, 1.25)*	1.46 (1.21, 1.77)*	1.74 (1.30, 2.31)*
	model 3	1.16 (1.02, 1.33)*	1.00 (reference)	0.94 (0.78, 1.13)	1.10 (0.95, 1.28)	1.23 (1.01, 1.48)*	1.37 (1.03, 1.82)*
	model 4	1.15 (1.01, 1.33)*	1.00 (reference)	0.95 (0.79, 1.15)	1.05 (0.90, 1.22)	1.13 (0.93, 1.38)	1.23 (0.92, 1.64)
	model 5	1.17 (1.02, 1.34)*	1.00 (reference)	0.95 (0.78, 1.14)	1.03 (0.88, 1.20)	1.11 (0.91, 1.35)	1.21 (0.90, 1.61)
Women, N		131,484	34,874	7,372	3,821	1,152	528
Observational period, yr		2.0 (1.4–2.8)	2.0 (1.3–2.4)	2.0 (1.2–2.3)	2.0 (1.2–2.3)	2.0 (1.2–2.4)	2.0 (1.2–2.7)
eGFR decline ≥ 30%, N (%)		2,757 (2.1)	667 (1.9)	109 (1.5)	75 (2.0)	27 (2.3)	13 (2.5)
HR (95% CI),	model 1	0.98 (0.90, 1.08)	1.00 (reference)	0.82 (0.66, 1.02)	1.12 (0.87, 1.44)	1.19 (0.78, 1.82)	1.19 (0.65, 2.15)
	model 2	0.96 (0.88, 1.05)	1.00 (reference)	0.82 (0.66, 1.02)	1.16 (0.90, 1.50)	1.28 (0.83, 1.96)	1.30 (0.72, 2.36)
	model 3	0.99 (0.90, 1.08)	1.00 (reference)	0.85 (0.68, 1.06)	1.12 (0.87, 1.44)	1.11 (0.72, 1.69)	0.93 (0.51, 1.69)
	model 4	0.98 (0.89, 1.08)	1.00 (reference)	0.86 (0.69, 1.07)	1.04 (0.81, 1.34)	1.00 (0.65, 1.54)	0.82 (0.45, 1.50)
	model 5	0.97 (0.88, 1.06)	1.00 (reference)	0.86 (0.69, 1.07)	1.02 (0.79, 1.32)	0.99 (0.64, 1.52)	0.80 (0.44, 1.46)

Median (25%–75%)
CI, confidence interval; eGFR, estimated glomerular filtration rate; HR, hazard ratio
*P < 0.05
Model 1, unadjusted.
Model 2, adjusted for age (year).
Model 3, adjusted for age (year), dipstick urinary protein (-, ±, 1+, 2+, and ≥ 3+), and eGFR (mL/min/1.73 m^2).
Model 4, adjusted for covariates in Model 3 and body mass index (kg/m2), mean arterial pressure (mmHg), hemoglobin A1c (%), high density lipoprotein cholesterol (mg/dL), uric acid (mg/dL), and current smoking.
Model 5, adjusted for covariates in Model 4 and current treatment for hypertension, dyslipidemia and diabetes, and history of cardiovascular disease.

Table S7. Baseline characteristics and outcomes of rare vs. occasional male drinkers (1:1) and rare vs. daily male drinkers with alcohol consumption of ≥ 60 g/day (4:1) matched by propensity score.

	Rare vs. occasional		Occasional vs. Daily, ≥ 60 g/day	
	Rare	Occasional	Occasional	Daily ≥ 60 g/day
Number	28,846	28,846	9,825	2,901
**Baseline characteristics**				
Age, year	65 (58–70)	65 (58–69)	61 (51–67)	60 (52–65)
BMI, kg/m^2	24.1 ± 3.3	24.0 ± 3.0	24.1 ± 3.4	24.0 ± 3.1
Current smokers, N (%)	6,432 (22.3)	6,481 (22.5)	3878 (39.5)	1,274 (43.9)
SBP, mmHg	129 ± 17	129 ± 16	133 ± 17	135 ± 18
DBP, mmHg	78 ± 11	78 ± 10	81 ± 11	82 ± 11
MAP, mmHg	95 ± 12	95 ± 11	99 ± 12	99 ± 12
HDL cholesterol, mg/dL	54 ± 13	54 ± 12	61 ± 16	64 ± 17
Hemoglobin A1c, %	5.4 ± 0.8	5.4 ± 0.8	5.3 ± 0.7	5.3 ± 0.8
Uric acid, mg/dL	5.9 ± 1.3	5.9 ± 1.3	6.3 ± 1.4	6.5 ± 1.5
eGFR, ml/min/1.73 m^2	73 (64–84)	73 (64–84)	77 (67–88)	78 (69–89)
Dipstick UP, -, N (%)	23,811 (82.5)	23,863 (82.7)	7,844 (79.8)	2,292 (79.0)
±	2,878 (10.0)	2,873 (10.0)	1,149 (11.7)	340 (11.7)
1+	1,406 (4.9)	1,391 (4.8)	550 (5.6)	176 (6.1)
2+	578 (2.0)	546 (1.9)	228 (2.3)	75 (2.6)
≥ 3+	173 (0.6)	173 (0.6)	54 (0.5)	18 (0.6)
Hypertension, N (%)	8,526 (29.6)	8,459 (29.3)	3,002 (30.6)	869 (30.0)
Dyslipidemia, N (%)	3,201 (11.1)	3,131 (10.9)	741 (7.5)	198 (6.8)
Diabetes, N (%)	2,179 (7.6)	2,156 (7.5)	612 (6.2)	176 (6.1)
CVD history, N (%)	3,287 (11.4)	3,260 (11.3)	608 (6.2)	167 (5.8)
**Outcomes during observational period**				
Observational period, yr	1.9 (1.1–2.5)	2.0 (1.1–2.5)	2.0 (1.1, 2.6)	2.0 (1.1, 2.6)
Difference in eGFR slope, ml/min/1.73m^2/year	-0.43 (-0.73, -0.13)*	1.00 (reference)	1.00 (reference)	-0.81 (-1.62, -0.01)*
eGFR decline ≥ 30%, N (%)	361 (1.3)	310 (1.1)	147 (1.5)	51 (1.8)
Unadjusted HR (95% CI)	1.18 (1.01, 1.37)†	1.00 (reference)	1.00 (reference)	1.18 (0.86, 1.62)

Mean ± SD; Median (25%–75%)

BMI, body mass index; CI, confidence interval; CVD, cardiovascular disease; DBP, diastolic blood pressure; eGFR, estimated glomerular filtration rate; HDL, high density lipoprotein; HR, hazard ratio; MAP, mean arterial pressure; SBP, systolic blood pressure; UP, urinary protein.

*P for interaction between alcohol consumption category and time < 0.05

†P < 0.05
